# Student-specific Factors Associated with Passing the USMLE Step 1 Examination Within an Institution-allotted Dedicated Study Period

**DOI:** 10.1007/s40670-025-02636-w

**Published:** 2026-01-16

**Authors:** Eva Spier, Emily Yamron, Bailey A. Frohlich, Rebecca Hyman, Michelle C. Gulfo, Daniel Guttman, Peter Ch’en, Scott M. Wilson, Juan Lin, Amanda C. Raff, Michelle A. Blackmore

**Affiliations:** 1 Albert Einstein College of Medicine, Bronx, NY USA; 2 Department of Medicine, Columbia University Medical Center, New York, NY USA; 3 Department of Internal Medicine, Yale New Haven Hospital, New Haven, CT USA; 4 Department of Medicine, Montefiore Medical Center, Albert Einstein College of Medicine, Bronx, NY USA; 5 Department of Medicine, Brigham and Women’s Hospital, Boston, MA USA; 6 Harvard Medical School, Boston, MA USA; 7 Department of Epidemiology and Population Health, Albert Einstein College of Medicine, Bronx, NY USA

**Keywords:** USMLE step 1, Step 1 dedicated, Passing step 1, Pass/fail

## Abstract

**Background:**

In 2022, USMLE Step 1 scoring was changed from numeric to pass/fail. The pass rate dropped from 95% in 2021 to 90% in 2023 among US allopathic MD students. Predictors for student success and how study habits have changed since the scoring change to pass/fail have not been well-studied. This study aims to evaluate potential factors associated with achieving an on-time pass for Step 1 (OTP, passing by the end of a six-week period) among students at a single US medical school.

**Methods:**

A survey evaluating study methods prior to and during the dedicated Step 1 period was sent to second-year medical students who took Step 1. Responses were linked to individual metrics, and logistic regression was used to evaluate each factor in relation to the probability of an OTP.

**Results:**

The survey response rate was 50.3% (*N* = 93). OTP was significantly associated with reviewing completed courses before the dedicated period (OR = 4.69, 95% CI 1.80-13.43), beginning a question bank before the dedicated period (OR = 4.11, 95% CI 1.62–10.86), all-preclerkship course average (OR = 1.32, 95% CI 1.17–1.54), highest MCAT score (OR = 1.18, 95% CI 1.03–1.39), first year CBSE score (OR = 1.15, 95% CI 1.12–1.34), second year CBSE score (OR = 1.21, 95% CI 1.12–1.34), and final practice exam score (OR = 1.39, 95% CI 1.18–1.77).

**Conclusions:**

These data highlight objective metrics and study habits associated with passing Step 1 on time and thereby may help identify at-risk students and inform institutional study recommendations.

**Supplementary Information:**

The online version contains supplementary material available at 10.1007/s40670-025-02636-w.

## Background

The United States Medical Licensing Examination (USMLE) is a three-part licensure examination required for all allopathic physicians during their medical training. USMLE Step 1, the first of the three parts, focuses on the basic sciences that are a foundation for medical knowledge and is typically administered during medical school [1]. Students received a numerical score on Step 1 until January 2022, when the test was changed to a pass/fail grading system. At the same time, the passing threshold was increased by 2 points [2]. Prior to this scoring change, the passing rates among United States medical degree (MD) students were 95% and 97% in the years 2021 and 2020, respectively [3]. By contrast, after the scoring change, the MD student passing rates were 91%, 90%, and 89% in 2022, 2023, and 2024 [3]. In summary, since the Step 1 scoring change, the Step 1 failure rate among US MDs has more than doubled. The decline in Step 1 passing rates is a pressing national issue in medical education. Some cite the change to pass/fail and increase in the passing threshold as key drivers of the change in pass rates, given the temporal association of the change in the grading system with the largest decline in pass rate. Other speculated causes include a lack of appropriate study materials for a pass/fail exam and the COVID-19 pandemic, with an associated increase in mental health struggles among students [4].

Most medical schools provide a dedicated period in the curriculum for students to study for Step 1. The duration of this period and timing relative to the clerkship year vary by institution. This study was conducted at an institution, which offers a two-week winter break followed by a six-week dedicated period for students in their second year of medical school, before the beginning of clerkships. In contrast, one study conducted at the University of Michigan Medical School assessed how many weeks students elected to use for their dedicated period. The study found that most chose greater than the allotted 8 weeks, and many rescheduled their exam to a later date than originally planned [5].

There have been many studies investigating factors that are associated with performance on Step 1. Studies prior to the pass/fail change assess factors associated with higher numerical score performance on the exam. Studies have consistently found that prior grades, including Medical College Admission Test (MCAT) scores, practice National Board of Medical Examiners (NBME) scores, Comprehensive Basic Science Examination (CBSE) scores, and preclinical course grades, are associated with higher Step 1 scores [6–11]. Studies have also found that completing an increased number of unique question bank practice questions and daily Anki use are associated with higher scores [9, 12–14].

While many studies investigate factors associated with strong performance on Step 1, there are no studies assessing factors associated with students’ readiness to take Step 1 within a given dedicated period, on schedule within the institution’s curriculum. Failure to take the Step 1 exam on time may result in various consequences, including delaying clinical rotations, delayed graduation, financial burden, and emotional distress [15]. A failure might also impact a student’s options for residency. With the focus now on passing the USMLE Step 1 exam, it is important for medical educators to recognize risk factors for students not passing the exam on time to allow for earlier intervention for at-risk students and to guide students towards passing on time. Further, medical schools can understand how their curriculum does or does not effectively support their students in taking and passing Step 1 on time. This study aims to identify student metrics and study habits associated with passing Step 1 by the end of a single institution’s six-week dedicated period and describe strategies that can be recommended to students and schools to promote success.

## Methods

### Study Design

This IRB-approved study was conducted using a cross-sectional survey. The development of the survey was conducted by a group of 8 medical students who have completed their USMLE Step 1 examination, in consultation with faculty involved in medical education. This survey was not formally validated. The study followed the Strengthening the Reporting of Observational Studies in Epidemiology (STROBE) cross-sectional checklist [16].

### Population

The population eligible to participate in this survey was students in the Class of 2026 (MD students) or Class of 2030 (MD/PhD students). These students represent the population of students at a single institution who took the USMLE Step 1 exam in the spring of 2024. Students were excluded if they took Step 1 before the institutional dedicated period (January 1 – February 11, 2024).

### Survey Design

This survey is part of a larger ongoing project to analyze outcomes over multiple cohorts; relevant data to this paper’s research aims have been included. The complete survey tool is available in the supplementary information.

### Survey Distribution

The survey was distributed to students via their institutional email by a member of the school administration not a part of the research team. Each student was sent an individualized link to the survey. The survey was available for completion between August 27, 2024 and October 8, 2024, for a total of six weeks, which was six months after completion of the dedicated period. Students who had not completed the survey via their individualized link after the initial email received reminder emails; a total of four reminder emails were sent during the period the survey was open. To incentivize responses, at the end of the survey administration period, 5 of the total respondents were randomly selected by the survey administrator to receive a single prize. Prizes ranged from school gear up to one $50 and one $100 Amazon gift card.

### Variable Definitions/Outcomes

#### Medical College Admission Test (MCAT) Score

The MCAT is a standardized exam administered to students who intend to apply to medical school. Scores fall along a normal distribution ranging from 472 to 528. Some students take the exam multiple times; in this study the highest score was used.

#### All-preclerkship Course Average

The mean score between 0 and 100 that students received across 17 courses taught during the preclerkship phase of the curriculum. This variable was selected because this institution has a pass/fail curriculum and, therefore, does not use GPA.

#### Comprehensive Basic Science Examination (CBSE)

The CBSE is a standardized exam which is similar in content to Step 1 and administered to students at the institution of interest once at the end of first year, and once in December of their second year immediately prior to the dedicated study period. The score is reported as percentage correct.

#### On-time Passing (OTP)

OTP was defined as passing Step 1 by the end of the six-week dedicated study period. Of note, the period followed a two-week winter vacation that some students used to study, resulting in an OTP after studying for more than six total weeks.

### Data Analysis

Anonymized data were linked by a school administrator to respondents’ Step 1 date, retake date if relevant, outcome for each Step exam, CBSE scores, final score for each class, number of failed courses, and MCAT score. Data were organized in Microsoft Excel and analyzed in R. Differences in prior exam scores and study behaviors were compared between groups using Wilcoxon rank-sum test or two-sample t-test for continuous variables and Chi-Squared test or Fisher’s exact test when appropriate. Variables with a p-value < 0.20 in the univariate logistic regression were considered for inclusion in the multivariable model. Multivariate logistic regression was performed to evaluate on-time passing as a function of demographic variables, prior exam scores, and study behaviors. ‏Bidirectional stepwise regression was undertaken to produce the multivariate model. The final model that was retained was produced via the step function in R using version 4.4.1.

## Results

### Demographics

185 students were invited to complete the survey. 94 students responded to the survey, and one student was excluded due to taking Step 1 before the start of the defined dedicated period. The median age of respondents was 25.0 years (IQR = 2.0 years), 67.7% were female, and 9.7% self-identified as underrepresented in medicine (URM) [17]. Full demographics as well as academic characteristics can be found in Table 1. There were no statistically significant differences in age, gender, or status of being URM between students who did and did not achieve an OTP.

**Table 1:** Demographic & academic characteristics

	Overall (*N* = 93)
Age	
Median (IQR)	25.0 (2.00)
Missing	2 (2.2%)
Gender	
Female	63 (67.7%)
Male	29 (31.2%)
Prefer not to answer	1 (1.1%)
Underrepresented in Medicine (URM)	9 (9.7%)
Average Grade, All Preclinical Coursework	
Median (IQR)	82.8 (7.70)
Highest MCAT score	
Median (IQR)	516 (3.00)
First year CBSE score	
Median (IQR)	45.0 (9.00)
Missing	1 (1.1%)
Second year CBSE score	
Median (IQR)	55.0 (12.0)
Missing	1 (1.1%)
Days per week studying during dedicated	
Median (IQR)	6.00 (1.00)
Missing	1 (1.1%)
Question bank use before dedicated	
No	33 (35.5%)
Yes	59 (63.4%)
Missing	1 (1.1%)
Peer tutor use	
No	24 (25.8%)
Yes	68 (73.1%)
Missing	1 (1.1%)
Studied over the summer after first year	
No	57 (61.3%)
Yes	36 (38.7%)
Reviewed completed courses prior to dedicated	
No	45 (48.4%)
Yes	48 (51.6%)

This table shows demographics and academic characteristics of the survey respondents. Continuous variables are reported as medians with interquartile range (IQR) in parentheses. Categorical variables are reported as numbers of relevant respondents with the percentage of the total indicated in parentheses

### Primary Outcome: On-time Passing

Factors associated with passing Step 1 within the 6-week dedicated period or achieving an on-time-pass (OTP), were assessed. The survey response rate was 50.3% (*n* = 93). There were 27 students (29%) who did not achieve an OTP. Three of these students failed on their first testing attempt; the remainder passed but required additional time to study after the end of the dedicated study period. The median number of days of studying was 42 days (IQR = 35–46 days *N* = 66) in the OTP group and 62 days (IQR = 55.75–68.5 days *N* = 27) in the non-OTP group (*p* = 2.0 × 10^− 10^). There were no significant differences in the number of days per week spent studying during the dedicated period between the OTP and non-OTP groups. In univariate analyses, average grade of all preclinical coursework, first year CBSE score, second year CBSE score, question bank use before dedicated, and reviewing completed courses prior to dedicated were all found to be significantly associated with OTP (Table 2). Studying for Step 1 over the summer, taking four or more consecutive days off during the dedicated study period, the number of blocks of questions per day, and working with a peer tutor were not found to be significantly associated with OTP. Of note, the response rate among those who failed Step 1 was not high enough for analysis of factors associated with passing versus failing.

**Table 2:** Univariate analysis of independent variables for achieving an on-time pass on step 1

	Not on-time pass (includes extended dedicated time and failures) (*N* = 27)	On-time pass (*N* = 66)	*P*-value
Age			
Median (IQR)	25.0 (1.75)	25.0 (2.00)	0.736
Missing	1 (3.7%)	1 (1.5%)	
Gender			
Female	17 (63.0%)	46 (69.7%)	0.269
Male	9 (33.3%)	20 (30.3%)	
Prefer not to answer	1 (3.7%)	0 (0%)	
Average Grade, All Preclinical Coursework			
Median (IQR)	79.4 (4.16)	84.0 (6.37)	< 0.001
Highest MCAT score			
Median (IQR)	516 (3.50)	516 (3.00)	0.102
First year CBSE score			
Median (IQR)	41.0 (6.00)	48.0 (10.0)	< 0.001
Missing	0 (0%)	1 (1.5%)	
Second year CBSE score			
Median (IQR)	49.0 (10.5)	58.0 (9.00)	< 0.001
Missing	0 (0%)	1 (1.5%)	
Days per week studying during dedicated			
Median (IQR)	6.00 (1.00)	6.00 (1.00)	0.894
Missing	0 (0%)	1 (1.5%)	
Question bank use before dedicated			
No	16 (59.3%)	17 (25.8%)	0.0055
Yes	11 (40.7%)	48 (72.7%)	
Missing	0 (0%)	1 (1.5%)	
Peer tutor use			
No	8 (29.6%)	16 (24.2%)	0.718
Yes	19 (70.4%)	49 (74.2%)	
Missing	0 (0%)	1 (1.5%)	
Studied over the summer after first year			
No	19 (70.4%)	38 (57.6%)	0.36
Yes	8 (29.6%)	28 (42.4%)	
Reviewed completed courses prior to dedicated			
No	20 (74.1%)	25 (37.9%)	0.00326
Yes	7 (25.9%)	41 (62.1%)	

This table shows univariate analyses between the on-time pass (OTP) and non-OTP groups. A p value <0.05 is considered significant. Continuous variables are reported as medians with interquartile range (IQR) in parentheses. Categorical variables are reported as numbers of relevant respondents with the percentage of the total indicated in parentheses

In multivariate logistic regression, the final model included second-year CBSE score, all-preclerkship course average, and beginning a question bank before the start of the dedicated study period. Second-year CBSE score and all preclerkship course average remained significant in the multivariate model. A summary of unadjusted and adjusted odds ratios can be found in Table 3. Odds of achieving an on-time pass (OTP) were higher among students with higher preclinical course averages (OR OTP vs. no OTP per one-point increase in course average 1.21, 95% CI 1.03–1.45), second year CBSE scores (OR OTP vs. no OTP per one-point increase in score 1.14, 95% CI 1.03–1.28), and question bank use before dedicated (OR OTP among those who used vs. did not use a question bank before dedicated 2.73, 95% CIT 0.77–10.17).

**Table 3:** Logistic regression analysis of factors associated with OTP

	Unadjusted OR (95% CI)	Adjusted OR (95% CI)	*P*-value in multivariate model
Average Grade, All Preclinical Coursework	1.32 (1.17–1.54)	1.21 (1.03–1.45)	0.02
Second year CBSE score	1.21 (1.12–1.34)	1.14 (1.03–1.28)	0.03
Question bank use before dedicated	4.11 (1.62–10.86)	2.73 (0.77–10.17)	0.12
First year CBSE score	1.15 (1.12–1.34)	–	–
Highest MCAT score	1.18 (1.03–1.39)	–	–
Reviewed completed courses prior to dedicated	4.69 (1.80–13.43.80.43)	–	–

This table shows the adjusted model with unadjusted odds ratios (ORs) for variables that were considered for the final model and adjusted ORs for variables that were retained after stepwise regression. A *p* value <0.05 is considered significant

### Reasons for Delaying Step 1 Administration

A subset of 27 surveyed students who delayed taking Step 1 beyond the dedicated study period indicated factors that contributed to their decision to delay taking the exam (Table 4). Of note, only two (7.4%) indicated that a single factor drove their decision; for the others, a variety of drivers impacted their choice. Most commonly, general concerns about burnout or mental health prompted a delay in sitting for Step 1 (*n* = 14, 51.8%). Other major drivers included not feeling comfortable sitting for the exam despite passing practice scores (*n* = 13, 48.1%), receiving only “low-passing” practice scores (*n* = 12, 44.4%), and not feeling that they had completed sufficient review either through question banks (*n* = 11, 40.7%), or content review (*n* = 10, 37.0%).

**Table 4:** Reasons why students did not take step 1 before the end of the institutional dedicated study period

No passing practice NBME scores	5
Low passing practice NBME scores	12
NBME scores were in passing range but not a score I felt comfortable with	13
USMLE denied requested testing accommodations	0
I have extra time for testing and needed additional time to study and/or complete practice tests	2
Test taking anxiety	7
Burnout/general mental health	14
I did not feel I had completed a sufficient portion of a question bank	11
I did not feel I had completed a sufficient amount of content review	10
Personal situation such as unexpected family or health event	6
Other	1

This table indicates reasons cited by 27 students for not taking Step 1 on time. The second column indicates the number of respondents who cited each reason. Note, students could select multiple reasons

## Discussion

The decline in Step 1 pass rates is a national issue in medical education. This single-institution survey study was designed to analyze factors associated with success on the exam within a given study period to help guide students and schools as they face this challenge. Factors associated with passing Step 1 by the end of a predetermined 6-week dedicated study period were evaluated.

The primary outcome of this study was on-time pass (OTP), or passing Step 1 by the end of a predetermined 6-week dedicated study period. Failure rate could not be assessed given the low response rate from students who failed on their first attempt. This was likely secondary to response bias and the fact that this was a study of one cohort at a single-institution study. Passing within a given time frame has not been assessed in prior studies, and factors contributing to delay and failure may overlap. Further, there are consequences for delaying the Step 1 exam that may have strong implications for a student’s overall academic success and emotional well-being. Insights from these findings based on OTP can be useful to institutions broadly, as their common goal is for all students to take the exam within a defined period and to be able to successfully advance through their curriculum on schedule.

Although this study is unable to determine causality, it identified correlations that are supported by other studies and may be helpful to students and medical educators. This study found that all-preclerkship course average, review of completed coursework during the preclerkship period, beginning a question bank during the preclerkship period, and exam scores, including MCAT and CBSE, were significantly associated with passing Step 1 by the end of the 6-week study period, or achieving an OTP. Further investigation of the reasons students delayed their test date revealed mental health concerns as the most cited factor. Many students who delayed the exam had achieved appropriate passing scores on their practice test but did not feel comfortable sitting for the test. In summary, the findings support that preparation in the pre-dedicated period, strong prior test performance, and healthy mental state during the dedicated period are important factors for achieving an OTP.

Most factors associated with OTP identified in this study took place in the pre-dedicated portion of medical school, suggesting that adapting study habits far before the formal dedicated period is important for Step 1 readiness. The association between preclerkship grade averages and Step 1 performance is well documented [8, 18–20]. While many institutions opt for a pass/fail curriculum, student preparation and performance remain critical for future success on board exams. Further, despite the current trend of third-party commercial resource use as part of a “parallel curriculum,” performance on institutional exams remains a strong predictor of Step 1 performance. While many studies have established an association between question bank usage and Step 1 performance, these studies do not differentiate between question bank usage during preclinical coursework and during a designated dedicated period [13]. Results from this study suggest not only the importance of question bank utilization, but early integration of question banks into studying.

Based on the findings of this study, the following recommendations for medical schools in the U.S can be made. Firstly, this data suggests an emphasis on early exam preparation, question bank usage, and the development of test-taking strategies. One possible modality may include the establishment or utilization of a structured peer-tutoring system early in the pre-dedicated period. Research also suggests that regular near-peer tutoring during preclinical years correlates with improved CBSE and USMLE Step 1 exam scores [21, 22]. This can help struggling students build this foundational knowledge that is crucial for an OTP. While this study did not find utilization of peer tutors to be associated with success, it is possible that potential associations were obscured via a form of “exposure misclassification.” Both students who met with a tutor once and those who frequently met with one indicated on the survey that they used peer tutoring support. One future direction to better understand the relationship of peer tutoring and on-time passing would be through analyzing the way students utilized this resource. Another way to build a foundation for strong exam performances is to integrate retrieval practice and spaced repetition into early coursework [23, 24]. Doing so ensures students enter the dedicated study phase with a solid foundation and better equips them to succeed on Step 1 and throughout their medical careers. Additionally, this research, in agreement with other studies, supports the correlation between CBSE scores and USMLE Step 1 passing [8, 25–27]. However, the literature has not yet identified a specific CBSE score indicative of passing. Though no formal recommendation can be made based on the quality of this evidence, medical school administrators may contemplate the use of CBSE scores to flag students who may be at risk of either failing Step 1 or not sitting for the exam according to the regular schedule. In this study, the median score was a 58 among the OTP group. Notably, this test was taken several weeks before Step 1 administration, and thus further improvement in practice test scores was expected. Institutions can use their CBSE data as guidance for identifying at-risk students before they begin a dedicated study period.

A final recommended area for investigation and intervention is reasons for exam delay. Students reported multifactorial reasons for delaying Step 1. Perceived poor mental health and lack of confidence despite receiving passing scores were the most cited reasons for delay. Mental health is an area of struggle for many medical students. A 2016 JAMA systematic review and meta-analysis found a prevalence of 27.2% of depression or depressive symptoms among medical students, greater than reported among the general population [28]. A 2022 meta-analysis found depression rates to be as high as 41% and anxiety to be 38% [29]. Mental health challenges are reported to be greater during periods of test preparation. Geraghty et al., describe how students taking their licensing exams experience an “exam mania,” or an increased period of pressure and burnout while studying for exams such as Step 1 [30]. Additionally, one study of student well-being during the dedicated period demonstrated that 81% of students had self-reported anxiety and 77% felt burned out [31]. The rising burden of poor mental health among medical students also correlated with the rise in Step 1 fail rates. Though no conclusions in this area can be drawn based on this single study, further research in this area is warranted. In particular, prospective research evaluating students for depression and anxiety using validated screening tools may help better characterize this concern. Perhaps, student mental health and wellbeing support can help students succeed during their preclerkship curriculum as well as during their formal dedicated periods [32, 33].

Limitations of this study include nonresponse bias and the potential for skewed results given the response rate of slightly greater than 50% of the eligible students. This survey was administered 6 months after the dedicated period, which increases the potential for recall bias. Finally, because this study was conducted at a single institution, there is a lack of generalizability to other schools, especially those that have a different curricular timing of their dedicated period. Additional research with longitudinal data and higher response rates across multiple institutions may address these limitations.

Further research in this area is warranted, including the evaluation of factors associated with passing versus failing Step 1. This survey highlighted the effect of poor student mental health on preparation for Step 1. Additional study in this area may guide initiatives for student wellbeing during dedicated and throughout medical school in general. Finally, future research may include collaboration with other institutions to assess factors associated with passing Step 1 more broadly, including at institutions where students take Step 1 after their major clinical year.

## Conclusions

In summary, this single-institution survey of medical students assessed factors associated with passing USMLE Step 1 by the end of a 6-week dedicated period. The strongest predictors of an on-time pass were pre-dedicated study habits and exam grades, suggesting the importance of early formation of strong study habits. Conversely, mental health struggles were the most cited reason students gave for postponing Step 1. This study identifies preparation strategies associated with passing Step 1 within a given time frame, supports the need for strong academic and mental health foundations, and highlights possible interventions for their potential to help students succeed.

## Supplementary Information

Below is the link to the electronic supplementary material.

Supplementary Material 1

Supplementary Material 2

## Data Availability

The survey can be found in the supplementary information. The data collected are not publicly available. The data that support the findings of this study may be available from the corresponding author upon reasonable request but will require permission from the medical school given the inclusion of data involving medical students. Data for this research include institutional data on student performance as well as survey data collected from students directly.

## Abbreviations

USMLE: United States Medical Licensing Examination
MD: medical degree
MCAT: Medical College Admission Test
NBME: National Board of Medical Examiners
STROBE: Strengthening the Reporting of Observational Studies in Epidemiology
OTP: on-time passing

## References

[1] Step 1 Exam Content | USMLE [Internet]. [cited 2025 Jul 14]. Available from: https://www.usmle.org/step-exams/step-1/step-1-exam-content

[2] USMLE Step. 1 Transition to Pass/Fail Only Score Reporting | USMLE [Internet]. [cited 2025 Jul 14]. Available from: https://www.usmle.org/usmle-step-1-transition-passfail-only-score-reporting

[3] Performance Data | USMLE [Internet]. [cited 2025 Jul 14]. Available from: https://www.usmle.org/performance-data

[4] English K. Assessing the impact of USMLE step 1 going pass-fail: a brief review of the performance data. Avicenna J Med. 2024;14(4):228–30. 40084226 10.1055/s-0044-1800830 PMC11896725

[5] Wang J, Crumbley ME, Nori S, Borah L, Holman E, Monrad SU. Many paths to the summit: survey of step 1 study methods with pass/fail scoring. Med Sci Educ. 2024;34(4):807–14. 39099856 10.1007/s40670-024-02072-2 PMC11296993

[6] Coumarbatch J, Robinson L, Thomas R, Bridge PD. Strategies for identifying students at risk for USMLE step 1 failure. Fam Med. 2010;42(2):105–10. 20135567

[7] Jeyaraju M, Linford H, Bosco Mendes T, Caufield-Noll C, Tackett S. Factors leading to successful performance on U.S. national licensure exams for medical students: a scoping review. Acad Med J Assoc Am Med Coll. 2023;98(1):136–48. 10.1097/ACM.0000000000004877 35857389

[8] Giordano C, Hutchinson D, Peppler R. A predictive model for USMLE step 1 scores. Cureus. 2016;8(9):e769. 27738569 10.7759/cureus.769 PMC5059149

[9] Guilbault RWR, Lee SW, Lian B, Choi J. Predictors of USMLE step 1 outcomes: charting successful study habits. Med Sci Educ. 2020;30(1):103–6. 34457646 10.1007/s40670-019-00907-x PMC8368851

[10] Keltner C, Haedinger L, Carney PA, Bonura EM. Preclinical assessment performance as a predictor of USMLE step 1 scores or passing status. Med Sci Educ. 2021;31(4):1453–62. 34457984 10.1007/s40670-021-01334-7 PMC8368122

[11] Liles J, Vuk J, Tariq S. Study habits of medical students: an analysis of which study habits most contribute to success in the preclinical years. MedEdPublish. 2018;7:61. 38089203 10.15694/mep.2018.0000061.1 PMC10712003

[12] Wothe JK, Wanberg LJ, Hohle RD, Sakher AA, Bosacker LE, Khan F, et al. Academic and wellness outcomes associated with use of Anki spaced repetition software in medical school. J Med Educ Curric Dev. 2023;10:23821205231173289. 37187920 10.1177/23821205231173289 PMC10176558

[13] Burk-Rafel J, Santen SA, Purkiss J. Study behaviors and USMLE step 1 performance: implications of a student self-directed parallel curriculum. Acad Med J Assoc Am Med Coll. 2017;92(11S Association of American Medical Colleges Learn Serve Lead: Proceedings of the 56th Annual Research in Medical Education Sessions):S67-74. 10.1097/ACM.0000000000001916 29065026

[14] Preparing to take the USMLE. Step 1: a survey on medical students’ self-reported study habits - PubMed [Internet]. [cited 2025 Jul 14]. Available from: https://pubmed.ncbi.nlm.nih.gov/25910497/10.1136/postgradmedj-2014-133081 25910497

[15] Toonkel RL, Pock AR, Hauer KE, Kogan JR, Seibert CS, Swan Sein A, et al. Stepping back: how should Pass/Fail scoring influence step 1 timing? Acad Med J Assoc Am Med Coll. 2025;100(2):137–43. 10.1097/ACM.0000000000005887 39316463

[16] STROBE [Internet]. [cited 2025 Jul 14]. Checklists. Available from: https://www.strobe-statement.org/checklists/

[17] AAMC [Internet]. [cited 2025 Jul 14]. FACTS Glossary. Available from: https://www.aamc.org/data-reports/students-residents/data/facts-glossary

[18] Investigating the Impact of Preparation Strategies on USMLE Step. 1 Performance - PubMed [Internet]. [cited 2025 Jul 14]. Available from: https://pubmed.ncbi.nlm.nih.gov/27500163/10.15694/mep.2015.004.0005 PMC4975378 27500163

[19] Zhang C, Rauchwarger A, Toth C, O’Connell M. Student USMLE step 1 preparation and performance. Adv Health Sci Educ Theory Pract. 2004;9(4):291–7. 15583484 10.1007/s10459-004-3925-x

[20] Parry S, Pachunka J, Beck Dallaghan GL. Factors predictive of performance on USMLE step 1: do commercial study aids improve scores? Med Sci Educ. 2019;29(3):667–72. 34457530 10.1007/s40670-019-00722-4 PMC8368955

[21] Khalil MK. Weekly near-peer tutoring sessions improve students’ performance on basic medical sciences and USMLE Step1 examinations. Med Teach. 2022;44(7):752–7. 35073221 10.1080/0142159X.2022.2027901

[22] Tanenbaum EJ, Johnson JH, Jordan E, Cottral J, Tenore C, Burton WB, et al. An effective Evidence-Based student run Near-Peer support group for the USMLE step 1 exam. Med Sci Educ. 2016;26(4):691–9.

[23] Upegui A, Awan OA. Spaced repetition in medical education: its importance and applications. Acad Radiol. 2024;31(12):5339–40. 39608867 10.1016/j.acra.2024.01.025

[24] Gilbert MM, Frommeyer TC, Brittain GV, Stewart NA, Turner TM, Stolfi A, et al. A cohort study assessing the impact of Anki as a spaced repetition tool on academic performance in medical school. Med Sci Educ. 2023;33(4):955–62. 37546209 10.1007/s40670-023-01826-8 PMC10403443

[25] Fagin AP, Engelstad ME, Markiewicz MR, Miloro M. Is there a correlation between comprehensive basic science examination and united States medical licensure examination step 1 performance among oral and maxillofacial surgery residents? J Oral Maxillofac Surg Off J Am Assoc Oral Maxillofac Surg. 2020;78(7):1054–60. 10.1016/j.joms.2020.02.006 32151653

[26] Glew RH, Ripkey DR, Swanson DB. Relationship between students’ performances on the NBME comprehensive basic science examination and the USMLE step 1: a longitudinal investigation at one school. Acad Med. 1997;72(12):1097–102. 9435717 10.1097/00001888-199712000-00022

[27] Guiot HM, Franqui-Rivera H. Predicting performance on the united States medical licensing examination step 1 and step 2 clinical knowledge using results from previous examinations. Adv Med Educ Pract. 2018;9:943–9. 30588149 10.2147/AMEP.S180786 PMC6298871

[28] Rotenstein LS, Ramos MA, Torre M, Segal JB, Peluso MJ, Guille C, et al. Prevalence of Depression, depressive Symptoms, and suicidal ideation among medical students: A systematic review and Meta-Analysis. JAMA. 2016;316(21):2214–36. 27923088 10.1001/jama.2016.17324 PMC5613659

[29] Peng P, Hao Y, Liu Y, Chen S, Wang Y, Yang Q, et al. The prevalence and risk factors of mental problems in medical students during COVID-19 pandemic: A systematic review and meta-analysis. J Affect Disord. 2023;321:167–81. 36341802 10.1016/j.jad.2022.10.040 PMC9613786

[30] Geraghty JR, Russel SM, Renaldy H, Thompson TM, Hirshfield LE. One test to rule them all: a qualitative study of formal, informal, and hidden curricula as drivers of USMLE “exam mania.” PLoS One. 2023;18(2):e0279911. 36735699 10.1371/journal.pone.0279911 PMC9897523

[31] Tackett S, Jeyaraju M, Moore J et al. Student well-being during dedicated preparation for USMLE Step 1 and COMLEX Level 1 exams. BMC Med Educ. 2022;22(1):16. 2022. 10.1186/s12909-021-03055-2 PMC8728922 34983481

[32] Lampe LC, Müller-Hilke B. Mindfulness-based intervention helps preclinical medical students to contain stress, maintain mindfulness and improve academic success. BMC Med Educ. 2021;21(1):145. 10.1186/s12909-021-02578-y. 33663478 10.1186/s12909-021-02578-y PMC7934360

[33] Wasson LT, Cusmano A, Meli L, et al. Association between learning environment interventions and medical student well-being: a systematic review. JAMA. 2016;316(21):2237–52. 10.1001/jama.2016.17573. 27923091 10.1001/jama.2016.17573 PMC5240821

