# Supplementary material for: Student-specific Factors Associated with Passing the USMLE Step 1 Examination Within an Institution-allotted Dedicated Study Period
Source: Med Sci Educ. 2026 Jan 16;36(2):611–8. doi: 10.1007/s40670-025-02636-w (PMC13197505; doi:10.1007/s40670-025-02636-w)
Supplement: Supplementary file 1 — Supplementary Material 1 [file 40670_2025_2636_MOESM1_ESM.docx]

**Article title:** Student-specific factors associated with passing the USMLE Step 1 examination within an institution-allotted dedicated study period

**Journal name:** Medical Science Educator

**Title:** Factors associated with on-time passing of the USMLE Step 1 examination

**Authors**: Eva Spier^1^*, Emily Yamron^1*^, Bailey Frohlich^1^, Rebecca Hyman^1^, Michelle C. Gulfo^1^, Daniel Guttman^1^, Peter Ch’en^1^, Scott Wilson^2,3^, Juan Lin^4^, Amanda Raff^5^, Michelle Blackmore^1^

*Indicates equal contribution.

^1^Albert Einstein College of Medicine, Bronx, NY

^2^Department of Medicine, Brigham and Women’s Hospital, Boston, MA

^3^Harvard Medical School, Boston, MA

^4^Department of Epidemiology and Population Health, Albert Einstein College of Medicine, Bronx, NY.

^5^Department of Medicine (Nephrology) Montefiore Medical Center, Albert Einstein College of Medicine, Bronx, NY

**Corresponding author:** Amanda Raff [araff@montefiore.org](mailto:araff@montefiore.org)

Start of Block: CONSENT

CONSENT **STEP 1 SURVEY (2024)** The goal of this survey is to assess Step 1 preparation methods and outcomes. This is a Research Study, and participation in this survey is voluntary. There is no anticipated direct benefit in participating, but we hope that responses will help guide future [INSTITUTION NAME] medical students in Step 1 preparation. Your responses will not be shared with outside bodies such as the LCME, and responses will not affect your academic standing. Once you begin the survey, the responses you submit before closing the browser will be included in our analysis. However, no questions are required and you are able to fully navigate back and forth through the survey to modify answers as you prefer. Only your final answers once you close the browser will be used. If you would like to void any or all of your responses, please contact [SURVEY ADMINISTRATOR] at [SURVEY ADMINISTRATOR EMAIL]. After you complete this survey, a member of the Office of Student Affairs who is not part of the research team will link your responses to other measures of academic performance, including preclinical grades and Step 1 pass/fail status. This person will de-identify the survey data BEFORE providing the data to the research team. All of the research team will be blind to the identity of the respondents. In exchange for your participation in this survey, you will be entered into a lottery for an Amazon gift card. **If you agree to participate in this survey, please click "Next" to continue**. Thank you for your participation.

End of Block: CONSENT

Start of Block: DEMOS

Q52 **DEMOGRAPHICS**

| 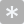 |
| --- |

DEDIC_START Please enter the date you began your dedicated period in MM/DD/YYYY format. *For reference, the CBSE was administered on December 14, 2023 and December 15, 2023, the final block 3 [INSTITUTION NAME]* *exam was December 19, 2023, and Winter Break ended on January 2, 2024. If unsure, round to the nearest Sunday.*

________________________________________________________________

GENDER What is your gender?

- Male (1)
- Female (2)
- Non-binary (3)
- Prefer not to answer (4)
- Other (5) __________________________________________________

| 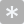 |
| --- |

AGE What is your age in years?

________________________________________________________________

RACE_ETHN What is your race and ethnicity? *Please select all that apply.*

- White (1)
- Hispanic or Latino (2)
- Black (3)
- Asian (4)
- American Indian or Alaska Native (5)
- Middle Eastern or North African (6)
- Native Hawaiian or Pacific Islander (7)
- Prefer not to answer (8)
- Other (9) __________________________________________________

PROGRAM Which [INSTITUTION NAME] training program are you a part of?

- MD (1)
- MSTP (2)

End of Block: DEMOS

Start of Block: PREDEDICATED

Q53 **PRE-DEDICATED PERIOD**

CLASS_RESOURCE For the following preclinical courses, please indicate which resources you used **at least weekly during the course**.

|  | [INSTITUTION NAME]-generated course materials (lectures, outlines, etc) (1) | Anki (2) | Third party question bank (e.g. UWorld, Amboss) (3) | Boards & Beyond (4) | Pathoma (5) | Sketchy (6) | Other (7) |
| --- | --- | --- | --- | --- | --- | --- | --- |
| Anatomy (1) |  |  |  |  |  |  |  |
| Genetics (2) |  |  |  |  |  |  |  |
| Pharmacology (3) |  |  |  |  |  |  |  |
| Immunology (4) |  |  |  |  |  |  |  |
| Biochemistry (5) |  |  |  |  |  |  |  |
| Hematologic System (6) |  |  |  |  |  |  |  |
| Pulmonary System (7) |  |  |  |  |  |  |  |
| GI/Liver System (8) |  |  |  |  |  |  |  |
| Cardiovascular System (9) |  |  |  |  |  |  |  |
| Renal System (10) |  |  |  |  |  |  |  |
| Nervous System and Human Behavior (11) |  |  |  |  |  |  |  |
| Endocrine System (12) |  |  |  |  |  |  |  |
| Infectious Disease (13) |  |  |  |  |  |  |  |
| Musculoskeletal System (14) |  |  |  |  |  |  |  |
| Reproductive Systems (15) |  |  |  |  |  |  |  |

OLD_REVIEW Did you study material from completed courses from the time you finished the course until the beginning of your dedicated period?  *This may include, but isn't limited to, not suspending Anki cards, watching videos ([INSTITUTION NAME]* *or third-party) about completed content, reviewing old notes, and doing questions*

- No (1)
- Yes (2)

OLD_REVIEW_1 Before dedicated began, what, if anything, did you do to revisit material from completed [INSTITUTION NAME] courses? Choose ALL that apply.

- I reviewed notes or review sheets from completed courses (1)
- I studied [INSTITUTION NAME] -generated materials (lectures, syllabi, etc.) from completed courses (2)
- I used third-party (e.g. Boards & Beyond, Pathoma, First Aid) resources to review content from completed courses (3)
- I did questions from a question bank (4)
- I used Anki and I un-suspended Anki cards from completed courses after first suspending them when the course finished (5)
- I used Anki and I continued reviewing Anki cards from completed courses without first suspending them when the course finished (6)
- I did not revisit material from completed courses prior to dedicated (7)
- Other (8) __________________________________________________

STUDY_START When did you begin a structured study schedule to study for Step 1? *A structured study schedule might include methods such as, but not limited to, setting aside time to review notes or Anki cards from completed courses for X minutes a day, doing X number of questions from a question bank weekly, creating a calendar and working through videos to review old content, etc.*

- Block 1 (first year, August-December) (1)
- Block 2 (first year, January-June) (2)
- Summer (first year, June-August) (3)
- Block 3 (second year, August-December) (4)
- Winter break/dedicated (second year, December-January) (5)

End of Block: PREDEDICATED

Start of Block: SUMMER STUDYING

Q55 **SUMMER STUDYING**

SUMMER_BIN Did you do any Step 1 preparation over the summer between the first and second years of medical school?

- No (1)
- Yes (2)

SUMMER_WK How many weeks during the summer did you do any preparation for Step 1?

|  | 0 | 1 | 2 | 3 | 4 | 5 | 6 | 7 | 8 |
| --- | --- | --- | --- | --- | --- | --- | --- | --- | --- |

| Weeks () | 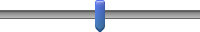 |
| --- | --- |

| 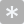 |
| --- |

SUMMER_HR To the nearest hour, on average how many hours did you study **each week** that you studied during the summer?

________________________________________________________________

SUMMER_RESOURCE What resources did you use over the summer? Please select all that apply.

- I reviewed notes or review sheets from completed courses (1)
- I studied [INSTITUTION NAME] -generated materials (lectures, syllabi, etc.) from completed courses (2)
- I used third-party (e.g. Boards & Beyond, Pathoma, First Aid) resources to review content from completed courses (3)
- I did questions from a third party question bank (4)
- I use Anki and I un-suspended Anki cards from completed courses after first suspending them when the course finished (5)
- I use Anki and I continued reviewing Anki cards from completed courses without first suspending them when the course finished (6)
- Other (7) __________________________________________________

SUMMER_QB On average, how many Question Bank questions did you complete **each week** over the summer? *If >200, please put 200.*

|  | 0 | 10 | 20 | 30 | 40 | 50 | 60 | 70 | 80 | 90 | 100 | 110 | 120 | 130 | 140 | 150 | 160 | 170 | 180 | 190 | 200 |
| --- | --- | --- | --- | --- | --- | --- | --- | --- | --- | --- | --- | --- | --- | --- | --- | --- | --- | --- | --- | --- | --- |

| 0 () | 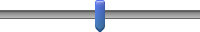 |
| --- | --- |

End of Block: SUMMER STUDYING

Start of Block: DEDICATED

| Page Break |  |
| --- | --- |

Q54 **DEDICATED PERIOD**

INTERRUPT After you began dedicated, was there any point at which you stopped your normal study routine for **4 or more consecutive days** between the start of your dedicated period and February 12, 2024 (the official end of dedicated)?

- No (1)
- Yes (2)

| Page Break |  |
| --- | --- |

| 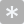 |
| --- |

INTERRUPT_TOT How many consecutive days did you take a break from studying between starting your dedicated Step 1 study period and taking your exam? *If you took multiple sets of breaks, calculate the total approximate number of days off.*

________________________________________________________________

DEDIC_DAYS On average, how many days a week did you study during your dedicated Step 1 study period?

|  | 0 | 1 | 2 | 3 | 4 | 5 | 6 | 7 |
| --- | --- | --- | --- | --- | --- | --- | --- | --- |

| Days () | 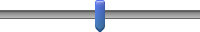 |
| --- | --- |

DELAY_BINARY Did you take Step 1 after the start of Transition to Clerkship (i.e. after February 12, 2024)?

- No (1)
- Yes (2)

DELAY_WHY If you took Step 1 after February 12, 2024, what factors contributed to when you chose to take Step 1? Choose **ALL** that apply!

- No passing practice NBME scores (1)
- Low passing practice NBME scores (2)
- NBME scores were in passing range but not a score I felt comfortable with (3)
- USMLE denied requested testing accommodations (4)
- I have extra time for testing and needed additional time to study and/or complete practice tests (5)
- Test taking anxiety (6)
- Burnout/general mental health (7)
- I did not feel I had completed a sufficient portion of a question bank (8)
- I did not feel I had completed a sufficient amount of content review (9)
- Personal situation such as unexpected family or health event (10)
- Other (11) __________________________________________________

End of Block: DEDICATED

Start of Block: DEDICATED HABITS

Q56 **DEDICATED STUDY HABITS**

DEDIC_QB When did you begin using a question bank such as UWorld or Amboss for intentional Step 1 studying?

- Block 1 (first year, August-December) (1)
- Block 2 (first year, January-June) (2)
- Summer between Blocks 2 & 3 (3)
- Block 3 (second year, August-December) (4)
- During my first week of dedicated (5)
- During my second week of dedicated or later (6)

DEDIC_OTH What type of studying were you doing prior to beginning to use a question bank during your dedicated Step 1 study period? *Check all that apply.*

- Content review using [INSTITUTION NAME] course materials (e.g. lectures or class notes) (1)
- Content review using third party resources (e.g. Sketchy, Boards & Beyond, First Aid) (2)
- Other (3) __________________________________________________

QB_PRIM What question bank did you primarily use during your dedicated Step 1 study period?

- UWorld (1)
- Amboss (2)
- Both UWorld & Amboss equally (3)
- Other (4) __________________________________________________

QB_DONE On average, how many *unique* questions (total from all question banks) did you **review** each day during your dedicated Step 1 study period?

- 0-19 (1)
- 20-39 (2)
- 40-59 (3)
- 60-79 (4)
- 80-99 (5)
- 100-119 (6)
- 120+ (7)

PEER Did you use a peer tutor to help prepare for Step 1 before or during your dedicated Step 1 study period? *This does not include attending the Step 1 kickoff event.*

- No (1)
- Yes (2)

PEER_IP How many times did you meet with a peer tutor in person or over zoom to prepare for Step 1?

- 0 (1)
- 1 (2)
- 2 (3)
- 3 (4)
- 4 (5)
- 5 (6)
- 6 (7)
- 7 or more times (8)

PEER_TXT Did you communicate with a peer tutor via text message, email, or other messaging during your dedicated Step 1 study period? *Please****do not include****messaging to coordinate zoom/in person meetings.*

- I messaged a peer tutor at least once weekly (1)
- I messaged a peer tutor less than once weekly (2)
- I did not message a peer tutor (3)

PEER_TOPIC In what areas did you receive peer tutoring? *Check all that apply.*

- Content review (1)
- Doing/reviewing questions, including practice exams (2)
- Test taking strategies (3)
- Making a study schedule (4)
- Stress management (5)
- Deciding when to take Step 1 (6)
- Managing mental health concerns (7)
- Managing work/life balance or unique personal circumstances (8)
- Other (9) __________________________________________________

End of Block: DEDICATED HABITS

Start of Block: SCORES

Q57 **PRACTICE TEST SCORES**

LAST_NBME_FORM What was the final NBME form you took before your exam?

- 25 (1)
- 26 (2)
- 27 (3)
- 28 (4)
- 29 (5)
- 30 (6)
- 31 (7)

LAST_NBME_SCORE What was the score you received on your final NBME? If you do not remember, please type "N/A"

________________________________________________________________

F120_BIN Did you take the free 120 before your exam?

- Yes, 2023 Free 120 (1)
- Yes, 2024 Free 120 (2)
- Yes, both (3)
- No (4)
- I don't remember (5)

F120_COUNTDOWN Approximately how many days before Step 1 did you take the free 120?  *If you took both the 2023 and 2024 free 120s, please respond using the date of the earlier free 120. If you do not remember, please write N/A.*

________________________________________________________________

F120_SCORE To the nearest 5 points, what was your score on the free 120? *If you took both the 2023 and 2024 free 120s, please respond using your first score.*

|  | 0 | 5 | 10 | 15 | 20 | 25 | 30 | 35 | 40 | 45 | 50 | 55 | 60 | 65 | 70 | 75 | 80 | 85 | 90 | 95 | 100 |
| --- | --- | --- | --- | --- | --- | --- | --- | --- | --- | --- | --- | --- | --- | --- | --- | --- | --- | --- | --- | --- | --- |

| Score () | 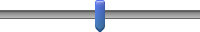 |
| --- | --- |

End of Block: SCORES

Start of Block: CANCEL

Q58 **FINAL QUESTION**

KILL_Statement All data collected in this survey will be analyzed, regardless of whether all questions were answered. If you wish to void any or all of your responses, please email [SURVEY ADMINISTRATOR] to do so ([SURVEY ADMINISTRATOR EMAIL]). Otherwise, to submit all your responses for analysis, click “Next”.

End of Block: CANCEL
