# Supplementary material for: Student-specific Factors Associated with Passing the USMLE Step 1 Examination Within an Institution-allotted Dedicated Study Period
Source: Med Sci Educ. 2026 Jan 16;36(2):611–8. doi: 10.1007/s40670-025-02636-w (PMC13197505; doi:10.1007/s40670-025-02636-w)
Supplement: Supplementary file 2 — Supplementary Material 2 [file 40670_2025_2636_MOESM2_ESM.docx]

**Title:** Student-specific factors associated with passing the USMLE Step 1 examination within an institution-allotted dedicated study period

**Authors**: Eva Spier^1^*, Emily Yamron^2*^, Bailey A Frohlich^3^, Rebecca Hyman^1^, Michelle C Gulfo^1^, Daniel Guttman^4^, Peter Ch’en^1^, Scott M Wilson^5,6^, Juan Lin^7^, Amanda C Raff^4^, Michelle A Blackmore^1^

**Supplementary Table 1:** Test statistics for continuous variables in Table 2 calculated with the Wilcoxon ranked sum test.

|  | **Z-score** |
| --- | --- |
| **Age** | 0.34 |
| **Average Grade, All Preclinical Coursework** | -4.77 |
| **Highest MCAT score** | -1.63 |
| **First year CBSE score** | -4.29 |
| **Second year CBSE score** | -4.98 |
| **Days per week studying during dedicated** | 0.13 |
|  |  |

**Supplementary Table 2:** Test statistics for categorical variable in Table 2 calculated with the chi squared test.

|  | **Chi Squared Statistic** |
| --- | --- |
| **Gender** | 2.62 |
| **Question bank use before dedicated** | 7.70 |
| **Peer tutor use** | 0.66 |
| **Studied over the summer after first year** | 0.83 |
| **Reviewed completed courses prior to dedicated** | 8.65 |
